# Addition of a Mixture of Plant Extracts to Diets for Growing-Finishing Pigs on Growth Performance, Blood Metabolites, Carcass Traits, Organ Weight as a Percentage of Live Weight, Quality and Sensorial Analysis of Meat

**DOI:** 10.3390/ani10071229

**Published:** 2020-07-20

**Authors:** José Luis Dávila-Ramírez, Lucas Lisandro Munguía-Acosta, Jubitza Guadalupe Morales-Coronado, Ana Delia García-Salinas, Humberto González-Ríos, Hernán Celaya-Michel, Jesús Sosa-Castañeda, Esther Sánchez-Villalba, Jesús Anaya-Islas, Miguel Angel Barrera-Silva

**Affiliations:** 1 Centro de Investigación en Alimentación y Desarrollo, A.C. (CIAD, A.C.), Carretera a la Victoria km. 0.6. Hermosillo, Sonora 83304, Mexico; jldavilaramirez@hotmail.com (J.L.D.-R.); hugory@ciad.mx (H.G.-R.); 2 Ciencia Aplicada para el Desarrollo Tecnológico, A.C. (CIADETEC, A.C.), Pedro Moreno # 24, Col. Centro Norte. Hermosillo, Sonora 83000, Mexico; 3 Departamento de Agricultura y Ganadería, Universidad de Sonora, Carretera a Bahía de Kino km. 21. Hermosillo, Sonora 83000, Mexico; lisandro14jul@gmail.com (L.L.M.-A.); yubytzam28@gmail.com (J.G.M.-C.); annagarcia@live.com (A.D.G.-S.); hernan.celaya@unison.mx (H.C.-M.); jesus.sosa@unison.mx (J.S.-C.); jesus.anaya@gmail.com (J.A.-I.); 4 Laboratory of Reproduction, Centre of Reproductive Biotechnology (CEBIOR-BIOREN), Faculty of Medicine, Universidad de la Frontera, Temuco 4780000, Chile; esthersanchezvillalba@gmail.com

**Keywords:** plant extracts, pork, heat stress, carcass characteristics, meat quality

## Abstract

**Simple Summary:**

The objective of this study was to assess the effect of adding a mixture of plant extracts on the productive performance, blood constituents, carcass characteristics, percentage relation between organ weight and live weight, quality, and sensory analysis of pork produced under heat stress conditions. The study was performed during the summer in the state of Sonora, Mexico. The production of pork is affected by heat stress conditions. The addition of plant extracts can reduce the negative effects of heat stress, because it is known that they improve health, growth, and some parameters of meat quality. Results indicate that the addition of plant extracts to diets of growing pigs improves the productive performance and carcass weight, without modifying blood constituents, and the quality and sensory attributes of the meat.

**Abstract:**

The effect of plant extracts (PE; artichoke, celery, beet, onion, garlic, spinach, avocado, oats, and parsley) in the diet of growing pigs under heat stress was investigated. Parameters included growth performance, blood constituents, carcass characteristics, organ percentage, quality and sensory appraisal of the pork. The study was performed during the Mexican summer, using 60 pigs. Treatments included the control, to which 0.1% PE, and 0.15% PE were added. The use of PE (0.1 and 0.15%) generated an increase in the average daily gain (ADG, by 10.0% for both treatments), and final live weight (LW, by 6.3% and 6.8%) (*p* < 0.05). The level of blood albumin at 95 kg was higher when supplementing with 0.1% PE (*p* < 0.05). At 120 kg LW, creatine kinase values showed a tendency to be different (*p* = 0.07). Carcass weight increased (*p* < 0.05) when adding PE. Supplementation with 0.1% PE decreased (*p* < 0.05) the red/green (a *) hue of the meat, whereas supplementation with 0.1% and 0.15% PE increased the yellow/blue (b *) hue (*p* < 0.05). The addition of PE improves pig growth performance, and carcass weight by reducing the negative effects of heat stress, without markedly modifying blood constituents, meat quality, and sensory attributes of the pork.

## 1. Introduction

Northwest Mexico is characterized by a hot climate, mainly during the summer months, when temperatures range between 26 °C and 48 °C in the shade, with an average of 33 °C. The thermoneutral zone for growing-finishing pigs is between 18 °C and 25 °C [1], and it is well known that temperatures above this thermoneutral zone induce heat stress [2], which has negative effects on feed intake, weight gain, feed conversion, and carcass characteristics [3,4]. Some plant extracts were shown to be beneficial in heat-stress poultry [5,6]. Research in pigs indicates that the use of plant extracts (PE) can improve productive performance [7,8], and digestibility of dry matter and protein [9,10]. PE possess anti-inflammatory effects [11], and antimicrobial effects for several pathogens [12]. Together with the improvements in pig health, improvements in the parameters of meat quality such as the oxidative stability, smell, and taste have been reported [13].

Some plant extracts individually induce positive effects on the animal. Celery (*Apium graveolens*) contains analgesic, and anti-inflammatory components [14], whereas garlic (*Allium sativum*) improves digestibility [15]. Aji et al. [16] indicate that onions (*Allium cepa*) can improve productive performance. Other extracts, like that of avocado (*Persea americana*) and parsley (*Petroselinum crispun*), have shown vasodilation [17] and antimicrobial [18] effects, respectively. Several plants extracts possess antioxidant properties including artichoke (*Cynara scolymus*) [19], oats (*Avena sativa*) [20], beet (*Beta vulgaris*) [14], and spinach (*Spinacea olearace*) [21].

Yang et al. [22] and Krotkiewski and Janiak [23] demonstrated that synergistic effects can be evoked when using mixtures of herbal components, both in vivo and in vitro. Currently, no information is available on the effect that the mixture of extracts of the aforementioned plants could have in supplementing pigs kept in commercial conditions during the summer. It is proposed that with the use of a mixture of different PE (celery, garlic, onion, avocado, parsley, artichoke, oat, beets, and spinach, contained in a commercial product, PROTORGAN^®^, Tlaquepaque, Jalisco, Mexico), benefits for the pig can be inferred.

The objective of the present study was to assess the effect of adding plant extracts to the grower and finisher diets of pigs on growth performance, blood constituents, carcass characteristics, organ weight as a percentage of live weight (LW), meat quality, and sensory analysis of pork meat in the growing and finishing stage, produced under hot climate conditions categorized as heat stress.

## 2. Materials and Methods

All procedures involving animal handling were conducted under the approved Mexican official guidelines for domestic animal care [24,25,26], and the study was approved by the University of Sonora Committee (Num.: USO313005360).

### 2.1. Animal and Housing Conditions

The study was performed in the porcine experimental unit of the Department of Agriculture and Livestock of the Universidad de Sonora. The study was performed in 60 pigs of commercial terminal crosses (Yorkshire × Landrace × Duroc), 30 males and 30 females, which were housed in an opened building similar to that used in commercial conditions in the growing to finishing periods, they were in individual pens, equipped with stainless steel feeders and nipple-type drinkers. Water and food were provided ad libitum. This study was performed in three phases of feeding: phase I (35 to 70 kg), phase II (70 to 95 kg) and phase III (95 to 120 kg live weight (LW). Pigs were individually identified with ear tags. Treatments were distributed based on weight and sex of the pigs, with 20 experimental units per treatment.

### 2.2. Source of the Plant Extracts (PE)

In this study, a commercial patented product (PROTORGAN^®^, from Guwlab, Tlaquepaque, Jalisco, Mexico) was used. The product contained extracts from the following plants: artichoke (*Cynara sculymus*), celery (*Apium graveolens*), beet (*Beta vulgaris*), onion (*Allium cepa*), garlic (*Allium sativum*), spinach (*Spinacea olerace*), avocado (*Persea americana*), oats (*Avena sativa*), and parsley (*Petroselinum crispun*).

### 2.3. Treatments

Pigs received one of three diets, provided as a meal: (1) control diet (CON), designed to satisfy the nutrients for high lean potential, high productivity, and used as a commercial ration in Hermosillo, Mexico during summer [27]; (2) 0.1% PE, which was the control diet +0.1% (as fed) of plant extracts, PROTORGAN^®^; and (3) 0.15% PE, which was the control diet +0.15% (as fed) of plant extracts, PROTORGAN^®^. Diets were formulated to be isonitrogenous and isocaloric (Table 1).

### 2.4. Growth Performance

The total weight of the feed provided, and the feed rejected in each pen was recorded daily during the study period. At the end of each phase, the average daily feed intake (ADFI) was calculated. Pigs were weighed individually at the same frequency as feed intake was measured, and these data were used to calculate the average daily gain (ADG), and feed conversion ratio (F:G) per phase. The experiment was finished once the pigs reached an average of 120 kg LW, when they were sent for slaughter.

### 2.5. Blood Metabolites

At the end of the last two phases, blood samples were taken from the 10 pigs in each treatment (approximately 7 mL of blood was collected via jugular venipuncture) using two Vacutainer tubes (Becton, Dickinson and Company, Franklin Lakes, NJ, USA). One tube contained ethylenediaminetetraacetic acid (EDTA), and the other contained no additive.

Blood from tubes containing EDTA was used for the hemogram blood test. This included the determination of red blood cells, hemoglobin, hematocrit, leukocytes, mean corpuscular volume (MCV), mean corpuscular hemoglobin (MCH), mean corpuscular hemoglobin concentration (MCHC), lymphocytes, monocytes, and platelets. This was performed using an automated Coulter Electronics × 10 system. The blood from tubes containing no additive was centrifuged at 10,000 rpm for 10 min, and the serum was separated and stored at −20 °C until it was assayed for blood parameters (glucose, total protein, albumin, creatine kinase [CK] and cortisol). Glucose, total proteins, albumin, and creatine kinase (CK) were determined using the appropriate laboratory kits, following the manufacturer’s instructions (RANDOX^®^ Manual). Cortisol was determined using ELISA methodology (Sigma-Aldrich^®^, St. Louis, MO, USA).

### 2.6. Slaughtering and Carcass Traits

Pigs were sent for slaughter at 120 kg LW. Pigs were slaughtered in the abattoir of the Departamento de Agricultura y Ganadería, of the Universidad de Sonora by trained personnel. Electrical stunning (head to head) using conventionally methods was applied before sticking and exsanguination, complying with the corresponding official Mexican standards [24]. The carcasses were individually weighed to record the hot carcass weight (HCW, carcass weight included head, skin and legs, without viscera, internal organs, flare fat, kidneys, diaphragm, genitals and tail). Carcasses were then chilled for 24 h at 4 °C to obtain the cold carcass weight (CCW), carcass lengths, dressing percentage. Carcass shrinkage was determined (HCW–CCW as a percentage of original HCW), fat thickness, the longissimus thoracis muscle (LM) area, and marbling (24 h postmortem) was determined. Fat thickness and the LM area (cm^2^) were measured at the 10th and 12th ribs. Marbling was also evaluated according to the guidelines of the United States Department of Agriculture. Finally, cooling loss and dressing values were calculated. The percentage of lean yield was calculated from equations as indicated in the corresponding Mexican Norm [28].

### 2.7. Percentage Relation between the Weight of Organs and Live Weight

The liver, heart, lungs, stomach, spleen, and kidneys were extracted from each animal, and the weight was recorded to calculate the organ weight as a percentage of LW for each organ.

### 2.8. Dissection of the Longissimus Thoracis (LT) Muscle and Sampling

After evaluating the carcasses, the LT of the left side was extracted (4th to 12th intercostal space) from 10 pigs per treatment. The meat samples were marked for identification, vacuum packed, and transported under refrigeration to the facilities of the Centro de Investigación en Alimentación y Desarrollo (CIAD) in the city of Hermosillo, Sonora, for the corresponding analyses.

After arrival at the laboratory, the samples were kept frozen at −18 °C. Before analysis, samples were thawed for 24 h at 4 °C, and then sectioned to carry out chemical, physicochemical, and sensory measurements. Sectioning of the samples was consistently performed following the same protocol, and in the same order from the caudal end (the 12th-rib interface) to the cranial part of the LT muscle. The first cut (2.0 cm) was used to determine the contents of moisture, protein, and intramuscular fat. Four pairs of samples (2.54 cm each) were used for the Warner–Bratzler shear force (WBSF) test, cooking loss determination, and sensory analysis. A 2.5 cm slice was used to analyze color, pH, and water-holding capacity (WHC). All measurements were recorded immediately after the samples were sectioned.

### 2.9. Meat Quality

#### 2.9.1. Chemical Composition

The moisture, intramuscular fat, and protein content of the meat was determined following AOAC methods [29] for moisture (method 950.46), fat (method 920.39), and protein (method 955.04). The results are expressed as a percentage of fresh weight.

#### 2.9.2. Physical Analysis

To measure the color parameters in the meat cuts, a Hunter Lab colorimeter was used. Color determination included the parameters L *, a *, b *, hue angle (HUE) using the formula tan^−1^ (b/a), and chroma (color saturation) using the formula Chroma = (a * + b *) ^½^. For measurements, the illuminator D65 with 10° was used in the colorimeter. Color determinations were performed on the surface of the cold samples (4–6 °C) in five sites on the surface of the muscle [30].

The pH was determined in cold meat samples at 4–6 °C, using a portable digital HANNA (Hanna Instruments, Woonsocket, RI, USA) potentiometer with a penetrating electrode provided with a HANNA HI 99163 thermometer. Measurements were performed in triplicate. 

Water Holding Capacity (WHC) was determined following the methodology of Sutton et al. [31]. The sample was placed in micro-nylon fabric, and introduced into a 50 mL propylene tube. The sample was centrifuged at 2800 *g* for 5 min at 4 °C. The WHC percentage was calculated according to the difference in weight of the sample before and after the centrifugation.

Texture (WBSF) measurements were made with a Texture Analyzer TAXT-Plus (Texture Technologies Corp., Scarsdale, NY, USA). To measure the cutting effort (CE) of the meat, 2.54 cm thick slices were cut, and then cooked in an electric fryer (Cook Master Ester, Model 3222-3) until reaching an internal temperature of 71 °C. Once cooked, samples were cooled to room temperature (25–30 °C) and then refrigerated at 4 °C for 24 h. To measure the cutting effort, the cooked sample was cut in pieces of 1 cm^2^ by 3 cm length along the direction of muscle fibers (10 times per cut). The CE was measured perpendicularly to the muscle fibers, using the Warner–Bratzler accessory cutter mounted on the TAXT-Plus texture meter. The WBSF values was expressed in kilogram-force. 

Cooking loss was determined by calculating the difference in weight of the sample before and after cooking it at an internal temperature of 71 °C in an electric fryer (Cook Master Ester, Model 3222-3), following the AMSA [32] technique.

### 2.10. Sensory Analysis

Sensory assessment was performed by a trained panel of 10 members [33]. The training of the assessment panel was achieved using the AMSA [32] methodology. One day before performing the sensory analysis, the meat cuts were removed from the freezer, and thawed at 4 °C for 24 h. The meat cuts were cooked using the same procedure described for the assessment of texture (WBSF). Each portion of meat was cut to a thickness of 1.27 × 1.27 cm. The trained panel (using a dim red light) assessed the cooked samples in terms of odor intensity, taste intensity, fatty sensation, tenderness, juiciness, and amount of connective tissue, using a structured linear scale of 10 cm. The value anchored to the left (0 cm) of the linear scale refers to a descriptive term that represents the lowest intensity of odor, taste, fat, tenderness, juiciness, and amount of connective tissue. The right end (10 cm) refers to the highest degree for each sensory characteristic. Two characteristics were assessed visually (total color and total appearance) on raw samples, under white light and using the same type of scale.

### 2.11. Statistical Analysis

Each pig was considered as an experimental unit. All data were explored prior to statistical analysis to discard any possible outliers. For the analysis of variance (ANOVA) of growth performance, analysis of blood metabolites and carcass characteristics, a random complete blocks design was used [34], with initial weight as a blocking factor. Data of the quality and sensory analysis of the meat were analyzed by ANOVA with a completely randomized design, data normality was performed using the Shapiro–Wilk test. The experimental diet was included in the model as the main factor. When statistical differences (*p* < 0.05) were observed among treatments, then means were compared using Tukey’s multiple rank test. Effects were accepted as different at *p* < 0.05, and tended to be significant at *p* < 0.10. All data were processed with SAS (ver. 9.1. SAS Inst. Inc., Cary, NC, USA) statistical software [35].

## 3. Results

### 3.1. Climatic Conditions

**Figure 1:**
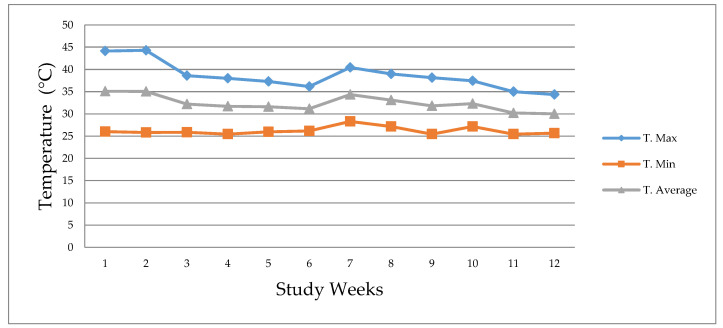
Maximum temperature (T. Max), minimum temperature (T. Min), and average temperature (T. Average).

The maxima, minimum and average temperature within the installation that housed the metabolic cages was recorded daily throughout the experimental period, utilizing a mercury thermometer (Figure 1). The thermometer was located in the installation at the height of the pigs. Temperatures ranged between 25.5 °C and 44.3 °C, average temperature of 32.4 °C. The relative humidity, was also recorded, and ranged between 19% and 56% with an average of 39%.

### 3.2. Growth Performance

Table 2 depicts the growth performance data in the three feeding phases. In Phase I (35 to 70 kg LW), there was only a difference in ADFI (*p* < 0.05), with a 15.0% increase when was comparing the 0.1% plant extract supplementation respect to the control diet. In the 70 to 95 kg stage there was a 14.3% increase in ADFI when supplementing with 0.1 plant extract as compared to the control diet, aside from a tendency in ADG to be different in PE-supplemented diets from the control diet (*p* < 0.10). Addition of 0.1% PE induced an increase (6.0%) in the final weight when compared to the control diet (*p* < 0.05). Regarding the last stage, 95 to 120 kg, addition of PE (0.1 and 0.15%) increased the ADFI in 17.2%, when compared to the control diet (*p* < 0.05). The ADG increased 18.2% when adding 0.15% of PE as compared to the control diet (*p* < 0.05). The final weight in this stage was higher in the animals whose diets was supplemented with 0.1% and 0.15% PE compared to the control diet, increases were of 6.3% and 6.8%, respectively (*p* < 0.05).

Analysis of the whole experimental stage (35 to 120 kg LW) revealed an improvement in ADG with either level of PE (0.1 or 0.15%) when compared to the control diet; increases were of 10.0% for both treatments (*p* < 0.05), without affecting the F:G (*p* > 0.05). There was an increase in feed intake (12.0%) when adding 0.1% PE, compared to the control diet (*p* < 0.05).

### 3.3. Blood Metabolites

Table 3 depicts the results of the blood metabolite analyses. At 95 kg LW, there were no statistical differences in most blood constituents (*p* > 0.05). The level of albumin was higher in the 0.1% PE diet, compared to the 0.15% PE and control diet, (6.7% for both treatments, *p* < 0.05). Creatine kinase (CK) values were not different (*p* = 0.1051). At 120 kg LW, there were no significant differences in the variables. CK values tended to be different among treatments (*p* = 0.07).

### 3.4. Carcass Traits

Carcass weight was increased when using either PE level (0.1% or 0.15% PE) compared to the control, both in the hot carcass (5.7% and 6.9%, respectively, *p* < 0.05), and the cold carcass (6.4% and 7.1%, respectively, *p* < 0.05, Table 4). Dressing percentage was not difference between groups (*p* > 0.05), whereas a reduction in the percentage of carcass shrinkage (*p* < 0.05) was observed when using 0.1% PE, as compared to the control diet. Length of the carcass, dorsal fat, depth, and area of the ribeye were similar among treatments (*p* > 0.05). Lean yield presented a tendency to be different (*p* < 0.10).

### 3.5. Organ Weight as a Percentage of Live Weight

No differences were observed among treatments when using PE at any concentration (*p* > 0.05) (Table 5).

### 3.6. Meat Quality

#### 3.6.1. Chemical Composition

Supplementation with either 0.1 or 0.15% PE did not induce changes in the content of moisture and protein as compared to the meat from non-supplemented animals (*p* > 0.05) (Table 6). Supplementation with 0.15% PE diminished intramuscular fat numerically as compared to the meat from non-supplemented animals (*p* > 0.05), but this was not significant.

#### 3.6.2. Physical Analysis

Physical parameters of the meat from pigs supplemented with PE are presented in Table 6. Addition of 0.1% and 0.15% PE induced a tendency to increase the values of L*, compared to the meat from non-supplemented animals (*p* < 0.10). Regarding parameter a* (red hue of the meat), the addition of 0.1% PE induced a close to 4.7% decrement in the values of the meat compared to the control group, and a 6.2% reduction compared with the 0.15% PE supplementation (*p* < 0.05). Addition of 0.1% and 0.15% PE increased parameter b* slightly (3.3% and 4.9%, respectively, *p* < 0.05) compared to the meat from control animals. The pH and WHC were not modified by the addition of PE (*p* > 0.05). Values of texture, a parameter assessed by measuring the cutting effort, were 9.9% higher in the meat from animals supplemented with 0.1% PE versus control animals, and 11.4% higher in animals supplemented with 0.15% of PE (*p* = 0.09).

### 3.7. Sensory Analysis

Table 7 depicts the sensory parameters of the meat from animals supplemented with PE. Addition of 0.15% PE produced meat cuts with higher visual coloration (*p* < 0.05). No changes were observed in overall color by adding PE (*p* > 0.05). The trained panel did not detect differences in the appearance of the meat cuts when sourced from animals supplemented with PE (*p* > 0.05). The meat cuts from animals supplemented with 0.15% PE had a better taste than those from control animals, and those supplemented with 0.1% of PE (*p* > 0.05). No differences were detected in the odor of meat supplemented with PE (*p* > 0.05). Addition of 0.1% of PE did not influence tenderness (*p* > 0.05). Juiciness of the assessed meat cuts was not different between groups (*p* > 0.05).

## 4. Discussion

### 4.1. Climatic Conditions

Animals have an optimal growth rate within their thermoneutral zone (TZ), which is defined as the range of environmental temperatures at which the normal maintenance, and productive functions of an animal in non-stressing conditions compensates for the heat lost to the environment without requiring a change in its metabolic heat rate [36]. For pigs in the growing and finishing stages, the TZ is in the range of 18 °C to 21 °C [37]. De Oliveira et al. [1] report that the thermoneutral conditions for the same stages (growing-finishing) are 18 °C to 25 °C. It is also known that temperatures above the TZ induce heat stress [2]. In the present study, temperatures ranged between 25.5 °C and 44.3 °C, with an average of 32.4 °C. These climatic conditions indicate that the animals were housed in pens at environmental temperatures very much above the optimal production conditions generating heat stress.

### 4.2. Growth Performance

Addition of PE induced an improvement in the ADG, ADFI and final LW, under heat stress conditions during the growing to finishing period. These results agree with other studies in which the addition of some type of plant extract or mixture of plant extracts have been assessed [5,6,38]. Frankič et al. [39] indicate that the addition of PE can impact physiological functions, and intestinal health, for a positive effect on growth performance. Despite the existing reports indicating the positive effect of adding PE to the diet of grower-finisher animals, there are reports that do not observe a positive effect on the productive performance [13]. The differences reported in the diverse studies could be due to the types of plant extracts added to the diet, as well as to different conditions in which the tests were performed, such as doses, health status, stage, ages at slaughtering, environment, among others. Despite of heat stress climatic conditions during the trial period, the use of PE cause an increment in the growth performance, a very favorable result for pig production in desert climates.

### 4.3. Blood Metabolites

Some blood metabolites are changed when the animals are under stress [40]. At 95 kg LW, the content of albumin, a metabolite related with stress in animals, increased when adding 0.1% PE, whereas the CK values tended to be different, probably due to the body weight reached by the pigs supplemented with PE, because the higher the weight of the animals, more susceptible they will be to heat [41]. Some reports indicate that the addition of a combination of plant extracts (buckwheat, thyme, curcuma, black pepper, and ginger) induces an increase in red blood cells, white blood cells, and lymphocytes in growing pigs [38], and in weanling pigs [42]. Increases in red blood cells of pigs in the growing-finishing stage when supplemented with *Coptis chinensis* extract [43] have been reported. No changes in these blood metabolites were reported. No modifications occurred in leukocytes and lymphocytes in those studies, which is similar to the results of this study. Halas et al. [44] and Li et al. [45] indicated an increase in lymphocytes when supplementing essential oils. All these parameters provide evidence that the addition of PE does not have any negative effect on blood metabolites; furthermore, it is important to note that the expected change in stress variables with increasing final LW was not perceived.

### 4.4. Carcass Characteristics

The weight of the carcasses, both hot and cold, improved with the addition of PE at the two analyzed concentrations (0.1% and 0.15%), which is related directly to the final LW obtained. Hossain et al. [46], and Rossi et al. [47] found no differences in the carcasses of pigs supplemented with plant extracts. Cullen et al. [48] found no differences in carcass characteristics using garlic and rosemary extracts for a 56-day period. Korniewicz et al. [49] reported that the addition of different levels of a mixture of plant extracts containing thymol, carvacrol, capsaicin, cinnamon aldehyde, eugenol, flavonoids, and essential oils to the diet of pigs of 20 to 100 kg LW did not induce differences in carcass quality variables. The discrepancy between the previously mentioned studies and our results are possibly due to the time of administration of the PE and the body weight at which the pigs were slaughtered at the end of testing.

### 4.5. Organ Weight as a Percentage of Live Weight

The use of growth-promoting molecules or certain diseases can cause the growth of an organ, which can influence the final weight and the yield. In the current study, the organ to LW percentage was not modified with any of the treatments. This agrees with Mahmood et al. [50], who indicated that the use of 0.5% garlic does not affect the weight of the heart, liver, and spleen. In contrast to our study, Dorhoi et al. [51] reported that the addition of a garlic extract to the feed of birds increases the weight of the thymus and spleen, together with an increment in the proliferation of lymphocytes and white blood cells. The differences reported in these studies could be due to the plant extract, doses, or health status.

### 4.6. Meat Quality

#### 4.6.1. Chemical Composition

Supplementation with PE have been reported to modify the quality of the meat of fattening animals, involving the fatty acid profile, and oxidative stability of the meat [52]. In the current study, supplementation with 0.15% PE numerically reduced the intramuscular fat content, whereas moisture and protein were modified only minimally, but these were non-significant. The moisture content ranged from 71.6% to 72.0%, whereas the protein content was close to 21.6% in all treatments, which are normal values in pork meat.

#### 4.6.2. Physical Analysis

Supplementation with 0.1% PE induced a small decrease in the red coloring of the meat, but the sensory panel did not detect this variation. Janz et al. [53], and Simitzis et al. [54] reported that color parameters of meat from pigs supplemented with plant extracts did not change. The addition of 0.1% PE tended to generate meat cuts with a greater cutting effort, a texture that was slightly detected by the trained panel, but again, not significantly so. In general terms of meat quality (Chemical Composition and Physical Analysis), it is evident that the addition of PE had no major effects on the pigs in the growing-finishing phases.

### 4.7. Sensory Analysis

Sensorial qualities (overall color, appearance, taste, odor, tenderness, juiciness, fat sensation and connective tissue) were not affected by the addition of the two PE concentrations, except in terms of visual color, where the panelists observed a higher intensity in the 0.15% PE diet compared with the control and 0.1% PE. Panelists categorized all meat cuts as close to a pale pink, the characteristic color of fresh pork meat. Hanczakowska et al. [13] reported a favorable effect on odor and taste of the meat sourced from pigs supplemented with PE, an effect that was attributed to an improvement in oxidative stability promoted by the plant extracts. Despite the favorable effect promoted by the addition of plant extracts on the meat, reports like the ones of Janz et al. [53] and Simitzis et al. [54] indicate that the addition of plant extracts to the diet of pigs does not modify the sensory characteristics of the meat.

The differences among the diverse mentioned studies can be due to the ingredients used in the diet, the environmental conditions, the dose of the added extract, time of extract administration, the health status of the animals, and the stage at which they were applied. It is important to continue with the assessment of plant extracts in other conditions that will improve the production and reduce the use of antibiotics.

## 5. Conclusions

The addition of plant extracts to the diets of pigs in the growing-finishing stage under heat stress improved growth performance, increased the carcass weight, and can be used as a strategy to minimize the negative effects of the summer, without marked effects on blood metabolites, percentage relation between organ weight and live weight, nor on the quality and sensory characteristics of the meat.

## Figures and Tables

**Table 1:** Composition of experimental diets for pigs in growing-finishing stages.

Ingredients (kg)	Treatments									
	Phase I			Phase II			Phase III			
	Control ^1^	0.1% PE ^2^	0.15% PE ^3^	Control ^1^	0.1% PE ^2^	0.15% PE ^3^		Control ^1^	0.1% PE ^2^	0.15% PE ^3^
Corn grain	68.85	68.75	68.70	76.10	76.00	75.95		77.00	76.90	76.85
Soybean meal	26.40	26.40	26.40	19.30	19.30	19.30		18.40	18.40	18.40
Fat	1.75	1.75	1.75	1.60	1.60	1.60		1.60	1.60	1.60
Plant extracts (Protorgan^®^)	0.00	0.10	0.15	0.00	0.10	0.15		0.00	0.10	0.15
Premix Initiation ^4^	3.00	3.00	3.00	-	-	-		-	-	-
Premix Growth ^4^	-	-	-	3.00	3.00	3.00		-	-	-
Premix Finish ^4^	-	-	-	-	-	-		3.00	3.00	3.00
Total	100.00	100.00	100.00	100.00	100.00	100.00		100.00	100.00	100.00
Chemical composition, as fed basis										
ME Cal. kg^−^^1, 5^	3360	3356	3354	3360	3356	3355		3361	3357	3356
Total lysine, %	1.18	1.18	1.18	0.98	0.98	0.98		1.01	1.01	1.01
Dig. lysine, %	1.05	1.05	1.05	0.87	0.87	0.87		0.90	0.90	0.90
Dry matter, %	88.98	88.97	88.98	88.76	88.74	88.77		88.72	88.74	88.71
Crude protein, %	18.27	18.27	18.26	15.48	15.4	15.47		15.12	15.12	15.12
Ca, %	0.70	0.70	0.70	0.66	0.66	0.66		0.67	0.66	0.66
Available P, %	0.31	0.31	0.31	0.28	0.28	0.28		0.26	0.26	0.26

^1^ Control = Control animals receiving only the basal diet, without plant extracts (PE) supplementation. ^2^ 0.1% PE = Animals receiving basal diet, supplemented with 0.1% PE. ^3^ 0.15% PE = Animals receiving basal diet, supplemented with 0.15% PE. ^4^ Premix provided the following per kg, with phases I, II, and III the same in vitamins and microminerals: 8000 IU vitamin A, 800 IU vitamin D3, 40 IU vitamin E, 3.5 mg vitamin K, 7 mg riboflavin, 20 mg pantothenic acid, 30 mg niacin, 30 μg vitamin B12, 550 mg choline, 64 mg Zn, 64 mg Fe, 4 mg Cu, 4 mg Mn, 0.4 mg Y, and 13 mg Se. Macrominerals, amino acids, and ractopamine were: phase I (initiation) 5.3 g Ca, 0.6 g P, 2.2 g lysine, 0.6 g methionine, and 0.6 g threonine; phase II (growth) 5.2 g Ca, 0.6 g P, 2.2 g lysine, 0.5 g methionine, and 0.5 g threonine; and phase III (finish) 5.4 g Ca, 0.4 g P, 2.8 g lysine, 0.6 g methionine, 0.3 g threonine, and 10 mg of ractopamine hydrochloride. ^5^ ME = Metabolizable energy (calories per kilogram).

**Table 2:** Growth performance according to phase.

Variable	Control ^1^	0.1% PE ^2^	0.15% PE ^3^	SEM ^4^	*p-*Value ^5^
Phase I, 35–70 kg					
ADFI ^6^, kg	2.0 ^b^	2.3 ^a^	2.1 ^ab^	0.04	0.04
ADG ^7^, kg	0.9	1.0	1.0	0.02	0.12
F:G ^8^, kg/kg	2.2	2.3	2.2	0.03	0.36
Initial weight, kg	35.6	35.6	35.6	0.07	0.96
Final weight, kg	68.5	71.7	70.7	0.64	0.13
Phase II, 70–95 kg					
ADFI ^6^, kg	2.8 ^b^	3.2 ^a^	3.0 ^ab^	0.05	0.02
ADG ^7^, kg	1.1	1.2	1.2	0.02	0.06
F:G ^8^, kg/kg	2.6	2.7	2.6	0.04	0.62
Final weight, kg	91.6 ^b^	97.1 ^a^	95.4 ^ab^	0.85	0.04
Phase III, 95–120 kg					
ADFI ^6^, kg	2.9 ^b^	3.4 ^a^	3.4 ^a^	0.06	<0.01
ADG ^7^, kg	1.1 ^b^	1.2 ^ab^	1.3 ^a^	0.03	0.03
F:G ^8^, kg/kg	2.5 ^b^	2.7 ^a^	2.5 ^b^	0.06	0.05
Final weight, kg	115.7 ^b^	123.0 ^a^	123.6 ^a^	1.17	0.01
Growing-finishing period 35–120 kg					
ADFI ^6^, kg	2.5 ^b^	2.8 ^a^	2.7 ^ab^	0.04	<0.01
ADG ^7^, kg	1.0 ^b^	1.1 ^a^	1.1 ^a^	0.01	0.01
F:G ^8^, kg/kg	2.4	2.5	2.4	0.03	0.10

^1^ Control = Control animals receiving only the basal diet without plant extracts (PE) supplementation. ^2^ 0.1% PE = Animals receiving the basal diet supplemented with 0.1% PE during the different phases. ^3^ 0.15% PE = Animals receiving the basal diet supplemented with 0.15% PE during the different phases. ^4^ SEM = Standard error of mean. ^5^ Probability values associated with the effects of plant extracts supplementation. ^6^ ADFI = Average daily feed intake. ^7^ ADG = Average daily gain. ^8^ F:G = Feed:gain ratio. ^a,^^b^ Within rows, means with different superscripts letter were significantly different (*p* < 0.05).

**Table 3:** Blood metabolites, sampled at 95 and 120 kg of average live weight.

Variable	Control ^1^	0.1% PE ^2^	0.15% PE ^3^	SEM ^4^	*p-*Value ^5^
	Analysis at 95 kg live weight				
Red blood cells, 1 × 10^3^ uL	4.8	4.7	4.7	0.14	0.89
Hemoglobin, g/dL	14.2	13.7	13.9	0.42	0.89
Hematocrit, %	41.8	40.5	41.1	1.24	0.90
Leukocytes, 1 × 10^3^ uL	13.6	13.3	13.6	0.51	0.97
MCV ^6^, fL	86.8	86.8	86.9	0.09	0.75
MCH ^7^, PG	29.4	29.4	29.5	0.04	0.83
MCHC ^8^, g/dL	33.9	33.9	33.9	0.02	0.21
Lymphocytes, 1 × 10^3^ uL	24.7	26.8	26.8	0.97	0.55
Monocytes, 1 × 10^3^ uL	4.9	5.2	4.2	0.34	0.41
Platelets, 1 × 10^3^ uL	317.2	280.1	293.5	12.48	0.42
Glucose, mg/dL	88.9	85.3	84.5	2.09	0.61
Total proteins, g/dL	5.6	5.5	5.5	0.04	0.45
Albumin, g/dL	3.0 ^b^	3.2 ^a^	3.0 ^b^	0.04	0.04
CK ^9^, u/L	174.5	250.7	181.2	16.89	0.11
Cortisol, μg/dL	2.8	3.3	4.2	0.30	0.14
	Analysis at 120 kg live weight				
Red blood cells, 1 × 10^3^ uL	4.5	4.5	4.7	0.11	0.77
Hemoglobin, g/dL	13.1	13.1	13.7	0.34	0.77
Hematocrit, %	38.9	38.6	40.4	1.00	0.79
Leukocytes, 1 × 10^3^ uL	16.7	17.1	15.1	0.44	0.25
MCV ^6^, fL	86.8	86.7	86.8	0.12	0.96
MCH ^7^, PG	29.4	29.4	29.5	0.04	0.70
MCHC ^8^, g/dL	33.9	33.9	33.9	0.02	0.30
Lymphocytes, 1 × 10^3^ uL	23.5	21.5	21.9	0.58	0.43
Monocytes, 1 × 10^3^ uL	5.9	7.0	7.6	0.38	0.26
Platelets, 1 × 10^3^ uL	293.9	250.3	293.9	11.71	0.42
Glucose, mg/dL	93.4	102.4	100.8	1.63	0.13
Total proteins, g/dL	7.1	7.2	6.8	0.20	0.72
Albumin, g/dL	3.0	3.1	3.2	0.06	0.48
CK ^9^, u/L	1710.4	616.8	1898.9	201.82	0.08
Cortisol, μg/dL	3.8	4.2	4.8	0.52	0.76

^1^ Control = Control animals receiving the basal diet without plant extracts (PE) supplementation. ^2^ 0.1% PE = Animals receiving the basal diet supplemented with 0.1% PE during the different phases of the growth period. ^3^ 0.15% PE = Animals receiving the basal diet supplemented with 0.15% PE during the different phases of the growth period. ^4^ SEM = Standard error of mean. ^5^ Probability values associated with the effects of PE supplementation. ^6^ MCV = Mean corpuscular volume. ^7^ MCH = Mean corpuscular hemoglobin. ^8^ MCHC = Mean corpuscular hemoglobin concentration. ^9^ CK = Creatine kinase. ^a,^^b^ Within rows, means with different superscripts letter were significantly different (*p* < 0.05).

**Table 4:** Carcass characteristics from growing to finishing pigs.

Variable	Control ^1^	0.1% PE ^2^	0.15% Pe ^3^	SEM ^4^	*p-*Value ^5^
HCW, kg ^6^	97.5 ^b^	103.1 ^a^	104.2 ^a^	0.96	0.01
Dressing, % (warm)	84.3	83.8	84.3	0.20	0.59
CCW, kg ^7^	96.4 ^b^	102.6 ^a^	103.2 ^a^	0.95	0.01
Dressing, % (cold)	83.3	83.4	83.5	0.20	0.85
Carcass shrinkage, %	1.1 ^a^	0.5 ^b^	0.9 ^ab^	0.09	0.02
Length of carcass, cm	99.5	102.2	101.1	0.59	0.18
Dorsal fat 10th, cm	1.4	1.7	1.5	0.06	0.27
Dorsal fat 12th, cm	1.0	1.2	1.2	0.05	0.17
Depth 10th, cm	7.7	7.8	8.0	0.11	0.53
Depth 12th, cm	7.3	7.3	7.5	0.12	0.72
Longissimus area 10th, cm	60.3	60.8	63.6	1.04	0.40
Longissimus area 12th, cm	64.7	63.8	67.7	1.21	0.42
Lean yield, %	53.9	53.0	53.2	0.13	0.06
Marbling	2.1	1.9	1.7	0.07	0.30

^1^ Control = Control animals receiving the basal diet without PE supplementation. ^2^ 0.1% PE = Animals receiving the basal diet supplemented with 0.1% PE during the different phases of the growth period. ^3^ 0.15% PE = Animals receiving the basal diet supplemented with 0.15% PE during the different phases of the growth period. ^4^ SEM = Standard error of mean. ^5^ Probability values associated with the effects of PE supplementation. ^6^ HCW = Hot carcass weight. ^7^ CCW = Cold carcass weight. ^a,^^b^ Within rows, means with different superscripts letter were significantly different (*p* < 0.05).

**Table 5:** Organ weight as a percentage of live weight (%).

Variable	Control ^1^	0.1% PE ^2^	0.15% PE ^3^	SEM ^4^	*p-*Value ^5^
Liver, %	1.4	1.3	1.3	0.02	0.61
Heart, %	0.4	0.3	0.4	0.01	0.15
Lungs, %	0.7	0.7	0.6	0.02	0.23
Stomach, %	0.6	0.5	0.5	0.01	0.16
Spleen, %	0.2	0.2	0.2	0.01	0.45
Kidneys, %	0.3	0.3	0.3	0.03	0.75

^1^ Control = Control animals receiving only the basal diet without plant extracts (PE) supplementation. ^2^ 0.1% PE = Animals receiving the basal diet supplemented with 0.1% PE during the different phases of the growth period. ^3^ 0.15% PE = Animals receiving the basal diet supplemented with 0.15% PE during the different phases of the growth period. ^4^ SEM = Standard error of mean. ^5^ Probability values associated with the effects of PE supplementation. ^a,^^b^ Within rows, means with different superscripts letter were significantly different (*p* < 0.05).

**Table 6:** Meat quality (chemical composition and physical analysis) of pigs supplemented with plant extracts (PE) during development and growing-finishing stages.

Variable	Treatments				
	Control ^1^	0.1% PE ^2^	0.15% PE ^3^	SEM ^4^	*p-*Value ^5^
Moisture, %	71.6	71.9	72.0	0.80	0.72
Fat, %	2.1	2.3	1.8	0.67	0.55
Protein, %	21.6	21.6	21.7	0.49	0.99
L*	53.3	55.8	55.7	1.37	0.10
a*	6.4^a^	6.1 ^b^	6.5 ^a^	0.77	0.01
b*	6.1 ^b^	6.3 ^a^	6.4 ^a^	0.22	0.03
HUE ^6^	47.6	46.8	47.4	0.69	0.56
CHROMA ^7^	8.8	8.7	9.1	0.29	0.25
pH	5.5	5.5	5.5	0.03	0.06
WHC ^8^	75.7	77.0	76.9	1.17	0.68
Cutting effort ^9^	7.1	7.8	7.0	0.27	0.09
Cooking loss	18.7	16.6	19.1	1.01	0.20

^1^ Control = Control animals receiving only the basal diet without PE supplementation. ^2^ 0.1% PE = Animals receiving the basal diet supplemented with 0.1% PE during the different phases of the growth period. ^3^ 0.15% PE = Animals receiving the basal diet supplemented with 0.15% PE during the different phases of the growth period. ^4^ SEM = standard error of mean. ^5^ Probability values associated with the effects of PE supplementation. ^6^ Hue angle = tan^−1(^b*/a*) × 57.29. ^7^ Chroma = (a* + b*)^½^. ^8^ WHC = water holding capacity. ^9^ cutting effort (WBSF) = Warner–Bratzler cutting effort. ^a,^^b^ Within rows, means with different superscripts letter were significantly different (*p* < 0.05).

**Table 7:** Sensory quality of meat from pigs supplemented with plant extracts (PE) during the growing-finishing stage.

Variable	Treatments				
	Control ^1^	0.1% PE ^2^	0.15% PE ^3^	SEM ^4^	*p-*Value ^5^
Visual color ^6^	1.6 ^b^	1.5 ^b^	1.9 ^a^	0.45	0.02
Overall color	7.0	7.1	7.0	0.07	0.93
Appearance	6.7	7.0	7.1	1.07	0.35
Taste	6.2	6.0	6.5	0.64	0.53
Odor	7.0	7.0	7.0	0.01	0.99
Tenderness	6.2	5.8	6.2	0.69	0.51
Juiciness	5.6	4.8	5.2	1.37	0.26
Fat sensation	1.4	1.2	1.4	0.21	0.81
Connective tissue	1.7	1.8	1.3	0.43	0.65

A 10 cm linear scale was used. Value of 0 (left) refers to a low grade of intensity in odor, taste, fat sensation, tenderness, juiciness, and connective tissue. Value of 10 (right) indicates the highest degree of acceptance for each sensory variable. ^1^ Control = Animals receiving only the basal diet without PE supplementation. ^2^ 0.1% PE = Animals receiving the basal diet supplemented with 0.1% PE during the different phases of the growth period. ^3^ 0.15% PE = Animals receiving the basal diet supplemented with 0.15% PE during the different phases of the growth period. ^4^ SEM = Standard error of mean. ^5^ Probability values associated with the effects of PE supplementation. ^6^ Visual color: 1 = Pale reddish-pink; 2 = pale pink; 3 = Pale greyish-pink. ^a,^^b^ Within rows, means with different superscripts letter were significantly different (*p* < 0.05).
